# A Systematic Review and Meta-Analysis of Prognostic Nomograms After UTUC Surgery

**DOI:** 10.3389/fonc.2022.907975

**Published:** 2022-07-01

**Authors:** Maximilian Pallauf, Frederik König, David D’Andrea, Ekaterina Laukhtina, Hadi Mostafaei, Reza Sari Motlagh, Fahad Quhal, Abdulmajeed Aydh, Takafumi Yanagisawa, Tatsushi Kawada, Pawel Rajwa, Lukas Lusuardi, Francesco Soria, Pierre I. Karakiewicz, Morgan Rouprêt, Michael Rink, Yair Lotan, Vitaly Margulis, Nirmish Singla, Evanguelos Xylinas, Shahrokh F. Shariat, Benjamin Pradere

**Affiliations:** ^1^ Department of Urology, Comprehensive Cancer Center, Medical University of Vienna, Vienna, Austria; ^2^ Department of Urology, University Hospital Salzburg, Paracelsus Medical University, Salzburg, Austria; ^3^ Department of Urology, University Medical Centre Hamburg-Eppendorf, Hamburg, Germany; ^4^ Institute for Urology and Reproductive Health, Sechenov University, Moscow, Russia; ^5^ Research Center for Evidence Based Medicine, Tabriz University of Medical Sciences, Tabriz, Iran; ^6^ Men’s Health and Reproductive Health Research Center, Shahid Beheshti University of Medical Sciences, Tehran, Iran; ^7^ Department of Urology, King Fahad Specialist Hospital, Dammam, Saudi Arabia; ^8^ Department of Urology, King Faisal Medical City, Abha, Saudi Arabia; ^9^ Department of Urology, The Jikei University School of Medicine, Tokyo, Japan; ^10^ Department of Urology, Okayama University Graduate School of Medicine, Dentistry and Pharmaceutical Sciences, Okayama, Japan; ^11^ Department of Urology, Medical University of Silesia, Zabrze, Poland; ^12^ Division of Urology, Department of Surgical Sciences, San Giovanni Battista Hospital, University of Studies of Torino, Turin, Italy; ^13^ Cancer Prognostics and Health Outcomes Unit, Division of Urology, University of Montréal Health Center, Montréal, QC, Canada; ^14^ Department of Urology, Pitié Salpétrière Hospital, Oncotype-Uro, Sorbonne University, Paris, France; ^15^ Department of Urology, University of Texas Southwestern, Dallas, TX, United States; ^16^ Departments of Urology and Oncology, The James Buchanan Brady Urological Institute, Johns Hopkins University School of Medicine, Baltimore, MD, United States; ^17^ Department of Urology, CHU Bichat, Paris, France; ^18^ Hourani Center for Applied Scientific Research, Al-Ahliyya Amman University, Amman, Jordan; ^19^ Department of Urology, Second Faculty of Medicine, Charles University, Prague, Czechia; ^20^ Department of Urology, Weill Cornell Medical College, New York, NY, United States; ^21^ Karl Landsteiner Insitute of Urology and Andrology, Karl Landsteiner Society, Vienna, Austria; ^22^ Department of Urology, La Croix du Sud Hôpital, Quint Fonsegrives, France

**Keywords:** UTUC, upper tract urothelial carcinoma, nomograms, prognostic models, oncologic outcome

## Abstract

**Background:**

Current guidelines recommend assessing the prognosis in high-risk upper tract urothelial carcinoma patients (UTUC) after surgery. However, no specific method is endorsed. Among the various prognostic models, nomograms represent an easy and accurate tool to predict the individual probability for a specific event. Therefore, identifying the best-suited nomogram for each setting seems of great interest to the patient and provider.

**Objectives:**

To identify, summarize and compare postoperative UTUC nomograms predicting oncologic outcomes. To estimate the overall performance of the nomograms and identify the most reliable predictors. To create a reference tool for postoperative UTUC nomograms, physicians can use in clinical practice.

**Design:**

A systematic review was conducted following the recommendations of Cochrane’s Prognosis Methods Group. Medline and EMBASE databases were searched for studies published before December 2021. Nomograms were grouped according to outcome measurements, the purpose of use, and inclusion and exclusion criteria. Random-effects meta-analyses were performed to estimate nomogram group performance and predictor reliability. Reference tables summarizing the nomograms’ important characteristics were created.

**Results:**

The systematic review identified 26 nomograms. Only four were externally validated. Study heterogeneity was significant, and the overall Risk of Bias (RoB) was high. Nomogram groups predicting overall survival (OS), recurrence-free survival (RFS), and intravesical recurrence (IVR) had moderate discrimination accuracy (c-Index summary estimate with 95% confidence interval [95% CI] and prediction interval [PI] > 0.6). Nomogram groups predicting cancer-specific survival (CSS) had good discrimination accuracy (c-Index summary estimate with 95% CI and PI > 0.7). Advanced pathological tumor stage (≥ pT3) was the most reliable predictor of OS. Pathological tumor stage (≥ pT2), age, and lymphovascular invasion (LVI) were the most reliable predictors of CSS. LVI was the most reliable predictor of RFS.

**Conclusions:**

Despite a moderate to good discrimination accuracy, severe heterogeneity discourages the uninformed use of postoperative prognostic UTUC nomograms. For nomograms to become of value in a generalizable population, future research must invest in external validation and assessment of clinical utility. Meanwhile, this systematic review serves as a reference tool for physicians choosing nomograms based on individual needs.

**Systematic Review Registration:**

https://www.crd.york.ac.uk/prospero/display_record.php?RecordID=282596, identifier PROSPERO [CRD42021282596].

## 1 Introduction

Upper tract urothelial carcinoma (UTUC) is a rare and biologically heterogeneous disease that accounts for less than five percent of all urothelial tumors (1). Given the disease’s heterogeneity, risk stratification leads the patient’s management.

Preoperative risk assessment guides the selection of treatment strategies in patients with localized disease, recommending kidney sparing surgery for low-risk (2) and radical nephroureterectomy for high-risk patients (3–6). Postoperative risk stratification decides on the administration of adjuvant chemotherapy and defines the follow-up strategy (7). For this purpose, the European Association of Urology guideline recommends using prognostic models (7). However, no specific model has been endorsed yet.

Improvements in postoperative patient counseling regarding adjuvant treatment are urgently needed. The POUT trial and Checkmate-274 provided evidence for a disease-free-survival benefit of adjuvant therapy (platin-based chemotherapy, nivolumab) (8–10). Conversely, IMvigor010, evaluating adjuvant immunotherapy with atezolizumab, failed to demonstrate any benefit (11). Patient risk stratification might explain these differences. The supplementary analysis of IMvigor010 showed that TNM-based risk stratification was insufficient in identifying patients in need of adjuvant treatment (12).

Among the various prognostic models, nomograms represent a user-friendly tool to predict a patient’s individual probability for a specific event, such as tumor recurrence or death (13–15). This information helps to individualize medical care and counsel patients based on evidence.

Over the past decades, various nomograms have been developed for postoperative UTUC patient counseling. However, there is no comprehensive overview to guide potential users regarding the utility or accuracy of these tools. It is, indeed, necessary to summarize the most reliable nomograms for clinical practice, identify those applicable for further research, and give suggestions for individual patient settings.

We performed a systematic review and meta-analyses on multivariable postoperative prognostic UTUC nomograms predicting oncological outcomes. Our secondary objectives were to outline and compare the nomograms, investigate their overall performance, identify the most reliable predictors, and provide physicians with a reference tool for clinical practice. 

## 2 Material and Methods

We performed this review following the recommendations of the Cochrane Prognosis Methods Group (16). The review protocol was prospectively registered in PROSPERO (registration number: CRD42021282596).

### 2.1 Search Strategy

We used the CHARMS checklist for systematic reviews of prediction modeling studies and the PICOTS scheme to define the review question (17). We searched for all studies that included UTUC patients (P), where a multivariable prognostic nomogram was investigated (I), to predict the oncologic outcome (O) (overall survival [OS], cancer-specific survival [CSS], recurrence-free survival [RFS], or intravesical recurrence [IVR]) in a one, three, or five years period (T), and that can be used after surgery or at a specific time point along the further course of the disease (postoperative) (S). Surgery was defined as any surgery intended to remove the tumor entirely.

Studies were eligible if (I) they matched the research question and (II) presented data on the development and internal or external validation of a multivariable prognostic nomogram. The studies also had to present data on (III) the nomogram’s prediction accuracy and calibration. (IV) Only full-text manuscripts published in English were included.

We searched the electronic databases Medline and EMBASE for studies published before December 2021. The search string used is listed on the PROSPERO website.

### 2.2 Data Collection

#### 2.2.1 Study Inclusion and Exclusion

**Figure 1:**
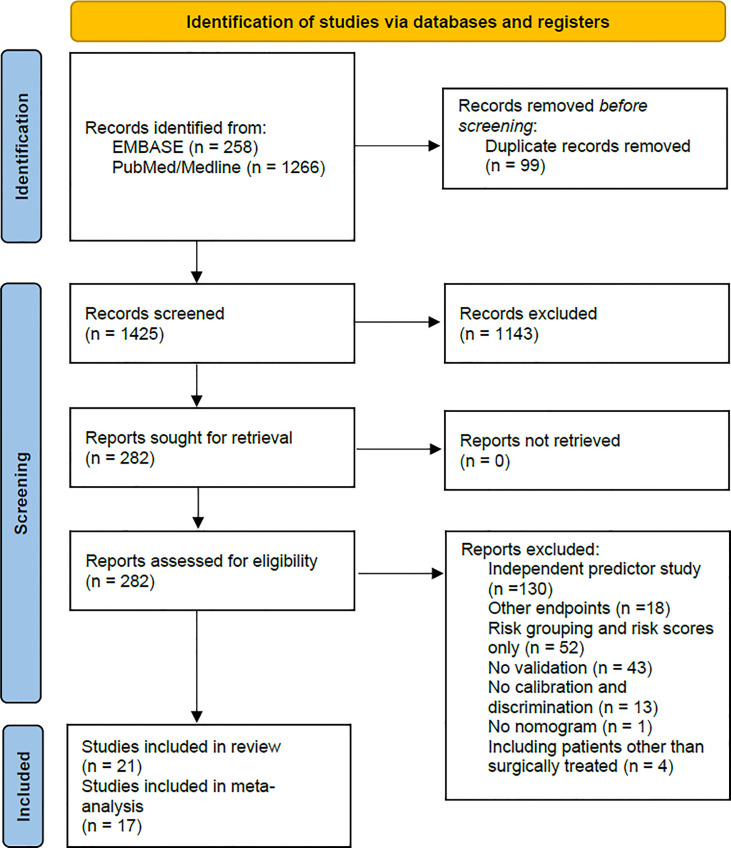
This figure shows the PRISMA flow chart of the study selection process. From: Page et al. (18).

Two reviewers independently screened the titles and abstracts to identify eligible publications and performed a full-text review based on the inclusion criteria. Disagreements between the two reviewers were resolved in consensus with the co-authors. **Figure 1** shows the PRISMA flow chart (18).

#### 2.2.2 Data Extraction and Management

Two reviewers independently extracted the data to a predefined datasheet, and a third reviewer verified the accuracy of the extraction process. We extracted data from the following domains: overall information, paper information, source data, participant information, outcomes to be predicted, model development, model validation, sample size, predictors, model performance, internal validation, and external validation. **Supplementary Table 1** lists the data extracted in detail.

#### 2.2.3 Model Performance Measures to be Extracted

We assessed the performance of each nomogram by extracting the measures for discrimination (c-Index with 95% confidence intervals [CIs] and standard error [SE], area under the receiver operating curve) and calibration (calibration plot interpretation, observed/expected ratio) presented without validation, and on internal and external validation. We assessed the independent effect of each predictor by extracting the hazard ratio (HR) or the coefficient Beta with 95% CIs and SE presented in the final model.

#### 2.2.4 Dealing With Missing Data

We calculated missing data of performance measures as recommended by Cochrane (16). The predictor’s Beta and Beta’s and HR’s SE were calculated with the given HR and the 95% CIs. Missing 95% CIs of HRs were calculated either with the SE or with the HR and its p-value (19). Missing 95% CIs and SE of the c-Index were calculated with the c-Index and the number of patients with and without events (20). All the corresponding authors were contacted in case of missing data.

### 2.3 Quality Assessment

#### 2.3.1 Assessment of Risk of Bias

Two reviewers independently assessed the risk of bias (RoB) of the included studies, using the dedicated Prediction model Risk Of Bias ASessment Tool (PROBAST) (21), which considered four potential sources of bias and three of applicability. The results of the PROBAST analysis were reported for each domain (bias: low risk, high risk, unclear; applicability: low concern, high concern, unclear), and an overall score for RoB and applicability was given.

#### 2.3.2 Assessment of Nomogram Heterogeneity

**Figure 2:**
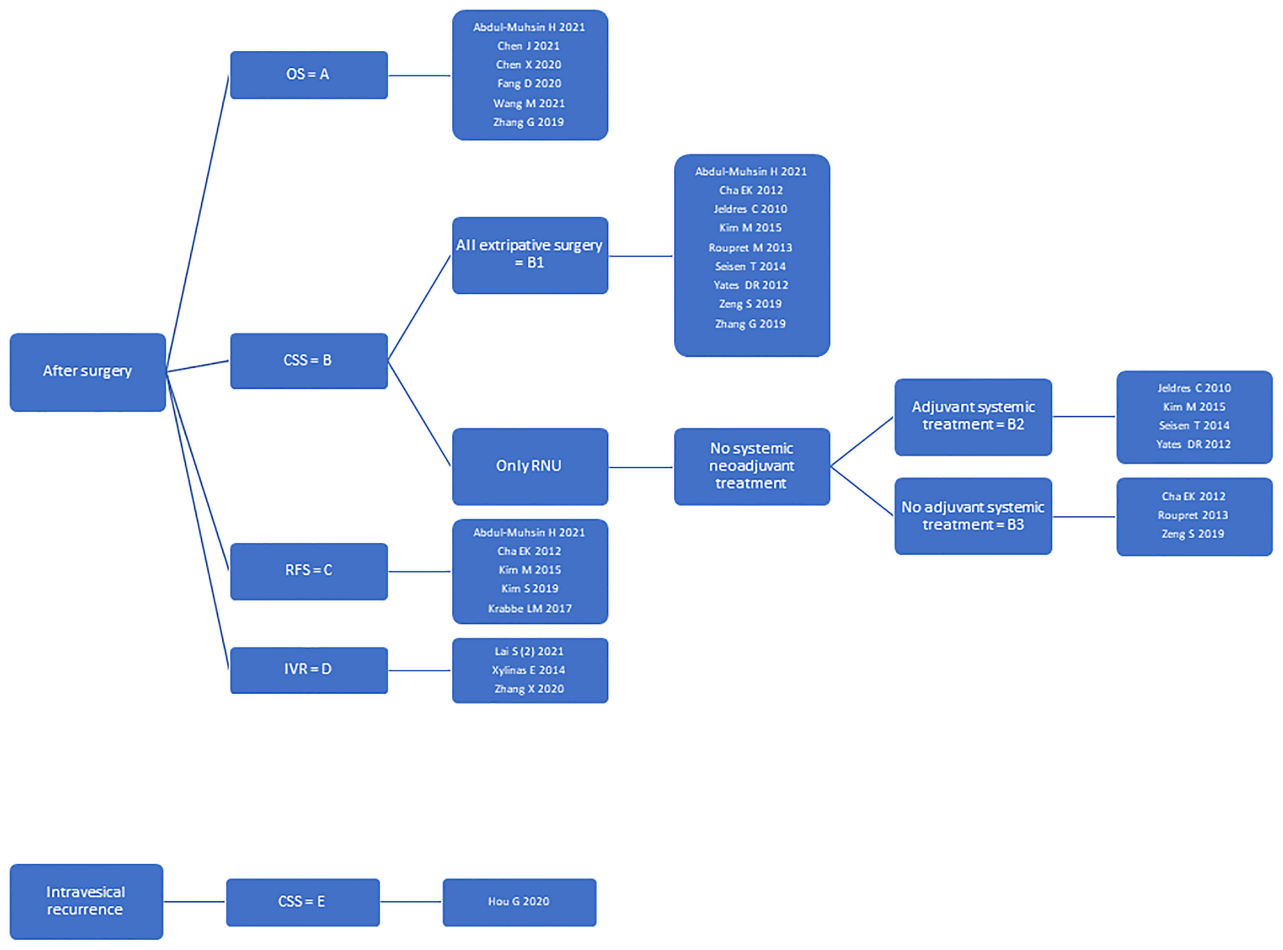
This figure shows the nomogram group stratification process. Further, it lists all nomograms within each group.

To account for nomogram heterogeneity, we stratified them into groups (A, B, C, D, E) regarding the purpose of use (after surgery or at the time of intravesical recurrence) and the outcomes to be predicted (OS or CSS or RFS or IVR). Group A included nomograms predicting OS, group B: CSS, group C: RFS, and group D: IVR after surgery. Group E included nomograms predicting CSS at the time of intravesical recurrence. We further stratified the nomograms from group B according to the surgical treatment (all types of surgery or radical nephroureterectomy only) and the patients’ baseline inclusion and exclusion criteria (neoadjuvant systemic treatment, adjuvant systemic treatment). Group B1 included nomograms for all types of surgery, whereas groups B2 and B3 included radical nephroureterectomy nomograms without systemic neoadjuvant treatment. Further, group B2 included nomograms with adjuvant and B3 without adjuvant systemic treatment. The nomogram groups were used for further statistical analysis. **Figure 2** highlights the nomogram group stratification process and lists the nomograms within each group.

### 2.4 Data Synthesis

#### 2.4.1 Summary of Nomograms

We summarized the key findings of the included studies by giving (I) general information on the publication, (II) the nomogram’s purpose of use, (III) predicted outcomes, (IV) validation types, (V) inclusion and exclusion criteria of the study, (VI) essential patient characteristics, (VII) nomogram predictors, (VIII) nomogram performance (without validation, on internal validation, on external validation), (IX) and the RoB and applicability of the publication/nomogram.

#### 2.4.2 Meta-Analysis Approach

We conducted the meta-analyses by nomogram groups due to the lack of validation studies and nomogram heterogeneity.

We investigated the overall performance of the nomogram groups by pooling the c-Index, which is a measure of discrimination accuracy. It accounts for censored data and is frequently used with survival data. Its value ranges between zero and one, with a value of 0.5 indicating prediction by chance (22). We set the cut-off values 0.6, 0.7, and 0.8 for moderate, good, and excellent nomogram discrimination accuracy. We included the c-Index that accounted best for the risk of overfitting in development studies: 1. Validation with an internal split cohort, 2. Validation with resampling by bootstrapping or cross-validation, 3. Development cohort without validation. We included the c-Index of all external validation studies.

We identified the most reliable predictors within each nomogram group by pooling Beta for predictors with a similar definition. The coefficient Beta is a measure of the predictor’s effect, and its value is independent of the measurement scale and therefore comparable among different variables. Positive values indicate a determinate effect, whereas negative values indicate a beneficial effect (23).

The number of three measurements (c-Index, Beta) was set as the lower limit for pooling. Therefore, we did not pool Beta within nomogram groups D and E and the c-Index for nomogram group E.

We used a Frequentist approach random-effects meta-analysis with the restricted maximum likelihood estimation and the Hartung-Knapp correction for calculating confidence intervals. If less than five studies were included, we additionally conducted a Bayesian approach random-effects meta-analysis. The meta-analyses results were plotted on a forest plot. The significance of the pooled summary estimate was assessed with the 95% CI, and the prediction interval (PI) verified its consistency. We used the statistical software R (v.4.0.5/2021) using the packages’ meta’ (24), ‘metafor’ (25), and ‘metamisc’ (20).

## 3 Results

### 3.1 Nomogram Search and Study Characteristics

From the 1524 records identified, we performed a full-text review of 282 articles and finally included 21 studies (26–46) for the systematic review and 17 studies (26–30, 32–35, 38–41, 43–46) for the meta-analyses. The full-text review excluded four studies that presented prognostic nomograms for UTUC patients receiving various treatments (surgery and/or radiotherapy and/or chemotherapy) (47–50). Nineteen studies presented nomogram development and internal validation data (26–35, 37, 39–46), of which two additionally presented external validation data of a separate nomogram (37, 44). Two studies presented external nomogram validation data only (36, 38). The development cohorts included 21,610 patients, and the internal split and external validation cohorts included 14167 patients. Patient data were collected in Asia in 11 (28–30, 33, 34, 36–38, 41, 44, 46), in North America in seven (26, 30–32, 41, 42, 45), in Europe in three (40, 42, 43), and worldwide in three (27, 35, 39) studies.

We identified 26 postoperative prognostic nomograms. All nomograms had been developed based on a cox-regression model. **Table 1** gives a detailed overview of the studies and nomograms, taking study inclusion and exclusion criteria into consideration. **Supplementary Table 2** lists the patient characteristics of development and validation studies in detail.

**Table 1:** This table summarizes the publications included in the systematic review, highlighting nomogram prediction outcome, nomogram validation, and patient inclusion and exclusion criteria.

GENERAL INFORMATION			ENDPOINTS								VALIDATION				INCLUSION / EXCLUSION CRITERIA											COMMENTS
First author	Year	Purpose (FS = following surgery, IV = intravesical recurrence, V = validation)	Overall Survival	OS timeframe (years)	Cancer Specific Surival	CSS timeframe (years)	Recurrence Free Survival	RFS timeframe (years)	Intravesical Recurrence	IVR timeframe (months)	Internal Validation Bootstrapp-/Resampling	Internal Validation Split Cohort / 2nd Cohort	External Validation Paper	External Validation Paper Author / Year	I/E Radical Nephroureterectomy	I/E Surgery other than RNU	I/E Systemic Neoadjuvant Treatment	I/E Systemic Adjuvant Treatment	I/E Intravesical Adjuvant Treatment	I/E Systemtic Palliative Treatment	I/E Badder Cancer~	I/E Contralateral UTUC~	I/E Other Malignancy or Systemtic Disaese	other important I/E	Nomogram groups	Y = yes N = no NI = no information ~if specifically named / otherwise other malignancy
Abdul-Muhsin H	2021	FS	Y	5	Y	5	Y	5	N		N	Y	N		I	E	I	I	NI	NI	NI	NI	NI	E: no currative intent	A, B1, C	
Cha EK	2012	FS	N		Y	2; 5	Y	2; 5	N		N	Y	Y	Zeng S 2019	I	E	E	E	NI	NI	E*	NI	NI		B1, B3, C	*no previous MIBC
Chen J	2021	FS	Y	3; 5	N		N		N		N	Y	N		I	E	E	I	NI	NI	NI	NI	E		A	
Chen X	2020	FS	Y	3; 5	N		N		N		Y	N	N		I	E	NI	NI	NI	NI	I	NI	E		A	
Fang D	2020	FS	Y	3; 5; 10	N		N		N		N	Y	N		I	I	E	E	NI	NI	NI	E	NI	E: solitary kidney	A	
Hou G	2020	IV	N		Y	1; 3; 5	N		N		Y	N	N		I	E	NI	NI	NI	NI	E*	E	NI	E: systemic recurrence before intravesical recurrence; more than one intravesical recurrence	E	*no bladder cancer before RNU
Jeldres C	2010	FS	N		Y	5	N		N		N	Y	N		I	E	NI	NI	NI	NI	NI	NI	NI		B1, B2	
Kim M	2015	FS	N		Y	2; 5	Y	2; 5	N		Y	N	N		I	E	E	I	NI	NI	E*	NI	E		B1, B2, C	*no previous MIBC
Kim S	2019	FS	N		N		Y	3	N		Y	Y	N		I	E	E	I	NI	NI	I	E	NI	E: previous or concurrent radical cystectomy	C	
Krabbe LM	2017	FS	N		N		Y	5	N		Y	Y	N		I	I	NI	NI	NI	NI	I	NI	NI	E: low grade UTUC	C	
Ku J	2013	V													I	E	E	I	NI	NI	NI	NI	NI	E: previous or concurrent cystectomy		
Lai S 1	2021	V													I	E	E	I	NI	NI	E*	E	NI			*no previous or conocmittant muscle invasive bladder cancer
Lai S 2	2021	FS	N		N		N		Y	12; 36; 60	Y	Y	N		I	E	NI	I	I	NI	E*	E	NI		D	*no synchronous baldder cancer, previous bladder cancer possible
Roupret M	2013	FS	N		Y	5	N		N		Y	Y	N		I	E	E	E	NI	NI	E*	NI	NI	E: pT0	B1, B3	*exclusion history of muscle invasive bladder cancer
Seisen T	2014	FS	N		Y	5	N		N		Y	Y	N		I	E	E	NI	NI	NI	E*	NI	NI	E: pTa, pT4, N 1-2, M1	B1, B2	*exclusion history of muscle invasive bladder cancer
Wang M	2021	FS	Y	1; 3; 5	N		N		N		N	Y	N		I	I	I	I	I	I	E	NI	NI	Nomogram I: HG only, Nomogram II: LG only	A	
Xylinas E	2014	FS	N		N		N		Y	3; 6; 12; 18; 24; 36	N	Y	Y	Lai S 1 2021 Lai S 2 2021	I	E	E	I	NI	NI	E*	NI	NI		D	*no previous muscle invasive bladder cancer and high grade non muscle invasive bladder cancer
Yates DR	2012	FS	N		Y	3; 5	N		N		Y	Y	Y	Ku J 2013	I	E	NI	NI	NI	NI	NI	NI	NI		B1, B2	
Zeng S	2019	FS	N		Y	3; 5	N		N		Y	Y	N		I	E	E	E	NI	NI	I	NI	NI		B1, B3	
Zhang G	2019	FS	Y	3; 5	Y	3; 5	N		N		N	Y	N		I	I	NI	NI	NI	NI	NI	NI	E		A, B1	
Zhang X	2020	FS	N		N		N		Y	24; 48	N	Y	N		I	E	E	I	I	NI	I	E	NI	E: pTa, pT4, N1-2, M1	D	

### 3.2 Nomogram Predictors

The median number of predictors used in the nomograms was 5.5 (Range 2-9). The most frequent predictors were pathological T-stage (n=21), age (n=17), pathological N-stage (n=16), tumor grade (n=12), and lymphovascular invasion (LVI) (n=11). In four nomograms, the reported HRs of the multivariable analysis and the assigned weights of the nomogram predictors did not match (42–44). **Supplementary Table 3** gives the predictors of each nomogram in detail. **Supplementary Table 4** summarizes the predictors most frequently used within each nomogram group.

### 3.3 Nomogram Performance Measures

The c-Index of development studies ranged from 0.657 (95% CI 0.560-0.755) (34) to 0.825 (95% CI 0.648-1) (41). Calibration plots of development studies showed moderate to good nomogram calibration, except for one nomogram (43).

The c-Index of external validation studies ranged from 0.683 (38, 42) to 0.742 (36, 43). A calibration plot of external validation was presented for only three nomograms (36–38, 42, 43). Nomogram calibration was good for two nomograms (37, 38, 42) but poor for the other (36, 43).

Neither development studies nor validation studies reported the observed/expected ratio. **Table 2** outlines the performance measures reported for the nomograms.

**Table 2:** This table gives a detailed overview of the performance of the nomograms (discrimination = c-Index, and calibration = interpretation of the calibration plot) on development and validation studies.

INFORMATION				Abdul-Muhsin H						Cha EK				Chen J		Chen X	Fang D	Hou G	Jeldres C	Kim M		Kim S
				2021						2012				2021		2020	2020	2020	2010	2015		2019
OUTCOME / GROUP				Overall survival		Cancer Specific Survival		Metasatasis Free Survival		Cancer Specific Surival		Recurrence Free Survival		Overall Survival		Overall Survival	Overall Survival	Cancer Specific Survival	Cancer Specific Survival	Cancer Specific Survival	Recurrence Free Survival	Recurrence Free Survival
				A		B1		C		B1, B3		C		A		A	A	E	B1, B2	B1, B2	C	C
**RESULTS**	**c-Index Development Paper¹**			0.784		0.714		0.753		0.815		0.768		0,804 (95%CI 0,713-0,895)		0.82	0,698 0,724	NI	0,753	0,802 (95%CI 0,752-0,851)	0,788 (95%CI 0,73-0,826)	0,657 (95%CI 0,560-0,755)
	**c-Index External Validation**			NI		NI		NI		0,69 0,7		NI		NI		NI	NI	NI	NI	NI	NI	NI
	**Calibration Plot Development Paper Interpretation Authors 3 years / 5 years (+/~/-)^1,2^**			NI / +		NI / +		NI / +		NI / +		NI / +		NI / +		+ / +	~ / NI	+ / +	NI / +	NI / +	NI / +	+ / NI
	**Calibration Plot External Validation Interpretation Authors 3 years / 5 years (+/~/-)²**			NI		NI		NI		NI / NI		NI		NI		NI	NI	NI	NI	NI	NI	NI
**INFORMATION**				**Krabbe LM**		**Lai S 2**		**Roupret M**		**Seisen T**		**Wang M**				**Xylinas E**		**Yates DR**	**Zeng S**	**Zhang G**		**Zhang X**
				**2017**		**2021**		**2013**		**2014**		**2021**				**2014**		**2012**	**2019**	**2019**		**2020**
**OUTCOME / GROUP**				**Recurrence Free Survival**		**Intravesical Recurrence**		**Cancer Specific Survival**		**Cancer Specific Survival**		**HG Group Overall Survival**		**LG Group Overall Survival**		**Intravesical Recurrence Full Model**	**Intravesical Recurrence Reduced Modell**	**Cancer Specific Survival**	**Cancer Specific Survival**	**Overall Survival**	**Cancer Specific Survival**	**No Intravesical Recurrence**
				**C**		**D**		**B1, B3**		**B1, B2**		**A**		**A**		**D**	**D**	**B1, B2**	**B1, B3**	**A**	**B1**	**D**
**RESULTS**	**c-Index Development Paper¹**			0,76 (±0,012)		NI		0,79 (95% CI 0,75-0,83)		0,8 (95%CI 0,76-0,84)		0,729 (95%CI 0,707-0,750) 0,763 (95% CI 0,656-0,869)		0,731 (95%CI 0,67-0,791) 0,825 (95%CI 0,648-1)		0.69	0.678	0.78	0,73 (95%CI 0,59-0,87)	0,702 (95%CI 0,684-0,720)	0,771 (95%CI 0,746-0,796)	0,678 (95%CI 0,583-0,772)
	**c-Index External Validation**			NI		NI		NI		NI		NI		NI		0.684	0.683	0.742	NI	NI	NI	NI
	**Calibration Plot Development Paper Interpretation Authors 3 years / 5 years (+/~/-)^1,2^**			NI / ~		+ / NI + / NI		NI / ~		NI / +		+ / +		+ / +		+ / NI	+ / NI	+ / -	~ / ~	+ / +	+ / +	+ / NI
	**Calibration Plot External Validation Interpretation Authors 3 years / 5 years (+/~/-)²**			NI		NI		NI		NI		NI		NI		+ / NI + / NI	+ / NI	- / -	NI	NI	NI	NI
							

¹We only report the c-Index/calibration plot accounting best for overfitting (split cohort validation > bootstrapping/resample validation > development cohort).

²If calibration plot was given but without interpretation from the authors, the reviewers interpreted the calibration plot. NI, no information given.

### 3.4 Nomogram Reference Tool

**Figure 2**, **Tables 1**, **2**, and **Supplementary Tables 2**, **3** represent a reference tool for postoperative UTUC nomograms. As a first step, **Figure 2** shall be used to identify nomograms predicting the outcome of interest. As a second step, **Table 1** needs to be checked for inclusion and exclusion criteria to be considered. If more than one nomogram is applicable, **Table 2** can be used to compare the nomogram’s diagnostic accuracy and calibration presented on internal and external validation cohorts. As the last step, **Supplementary Tables 2**, **3** can be checked to evaluate whether the patient’s characteristics align with the nomogram’s development and validation cohort and whether the predictors are readily available. Using this stepwise approach, physicians can choose a nomogram that fits the individual patient’s needs.

### 3.5 Risk of Bias Assessment of Included Studies

For development studies, overall RoB was high in 100% of the studies. RoBs mainly were due to inconsistencies in the analysis (100%) and participants domains (54%). Most predictors were selected based on the results of a univariable analysis, and the complexity of the data was not considered. Moreover, the data source and patient inclusion and exclusion criteria had a high RoB. Overall applicability of development studies was unclear in 42% of the studies, mainly due to inconsistencies in the participants (31%) and predictors (11%) domains.

For validation studies, overall RoB was high in 100% of the studies. RoBs mainly were due to inconsistencies in the analysis (60%) and participants (60%) domains. All studies validated the nomograms with retrospective cohorts, and in most cases, this resulted in a high RoB. Furthermore, most validation studies did not report handling of missing data and did not update the nomograms. Overall, the applicability of the validation studies was good.

**Figure 3:**
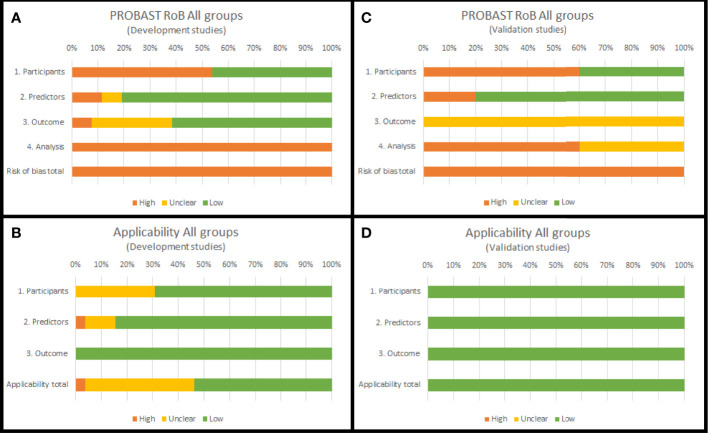
PROBAST summary (RoB domains and applicability domains) for all nomogram development **(A, B)** and validation studies **(C, D)** included in this systematic review.

**Figure 3** summarizes the PROBAST assessment of nomogram development and validation studies. **Supplementary Table 5** reports the PROBAST assessment of all nomograms.

### 3.6 Meta-Analyses

#### 3.6.1 Pooled Predictor Coefficient Beta

Advanced pathological T-stage (≥ pT3) was a significant and consistent negative predictor (Beta summary estimate with 95%CI and PI > 0) of OS (Nomogram group A). Age, pathological T-stage (≥ pT2), and LVI were significant negative predictors (Beta summary estimate with 95%CI and PI > 0) of CSS (Nomogram group B1). LVI was a significant and consistent negative predictor (Beta summary estimate with 95%CI and PI > 0) of RFS (Nomogram group C). CSS subgroups B2 and B3 had no significant and consistent predictors. The maximum number of coefficients pooled per predictor was six. See **Supplementary Figures 1**–**5**.

#### 3.6.2 Pooled c-Index

**Figure 4:**
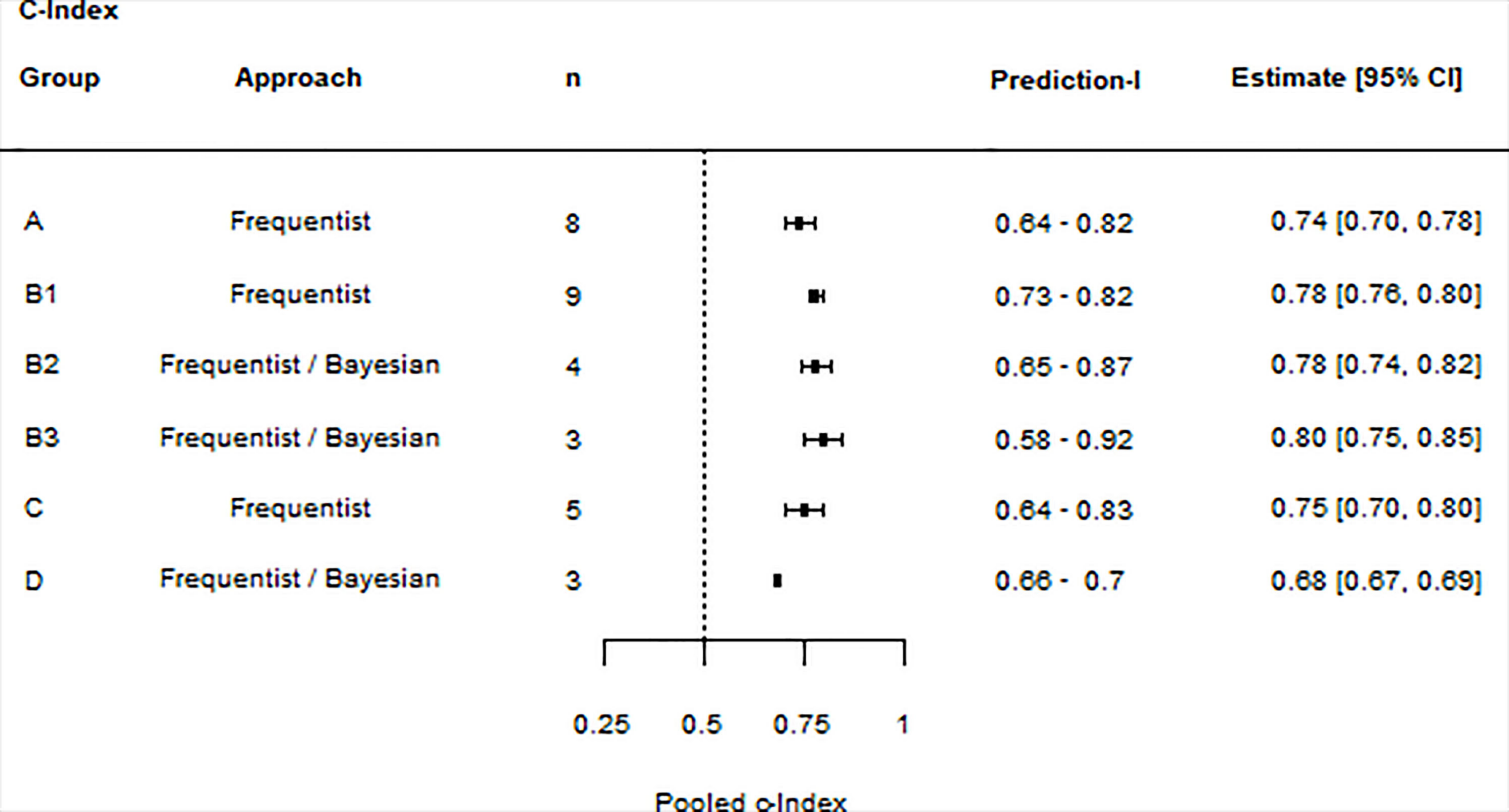
Summary forest plot of c-Index meta-analyses: The forest plot lists the results of individual meta-analyses. For each meta-analysis, the nomogram group, the statistical approach, the number of values included (n), the prediction interval (lower limit – upper limit), and the c-Index summary estimate (estimate and 95% CIs) are given.

Nomograms predicting OS (Nomogram group A), RFS (Nomogram group C), and IVR (Nomogram group D) had a significant and consistent moderate discrimination accuracy (c-Index with 95%CI and PI > 0.6). Nomograms predicting CSS (Nomogram group B1) had a significant and consistent good discrimination accuracy (c-Index with 95%CI and PI >0.7). CSS subgroup B2, but not B3, had a significant and consistent moderate discrimination accuracy (c-Index with 95%CI and PI >0.6). The maximum number of c-Indexes pooled per nomogram group was nine. See **Figure 4**.

## 4 Discussion

The discrepancy between nomogram development and external validation studies was high. Out of 26 nomograms, only four had been externally validated with a maximum of two validation cohorts. Indeed, the lack of external validation is a significant drawback for generalizability and discourages the uninformed use of nomograms in clinical practice. A common problem seen for prognostic model studies, as their five-year validation rate was shown to be only 16% (51).

We found that patients’ baseline characteristics varied widely between the studies. Patients had been recruited across different periods and continents and received varying treatment regimens. The increasing incidence of primary metastatic disease might have influenced nomograms’ predictions (52, 53). However, oncologic outcomes remained unchanged throughout the last decades (52). Further, the extent of surgical treatment can impact oncologic outcomes, either by increasing the risk of incomplete tumor resection or by selecting patients with favorable pathology (2). This is of particular importance, as previous studies highlighted the inaccuracy of preoperative staging (54). Even patients with relatively small tumors are at risk of muscle-invasive or even non–organ-confined disease (55). Similarly, excluding perioperative chemotherapy might select patients with favorable pathology or those unfit to receive the treatment. More importantly, it directly impacts the outcome (56). Heterogeneity in patient characteristics and its impact on oncologic outcomes is also a major drawback for the generalizability of these nomograms.

There were critical methodological weaknesses in the nomogram development studies. The data source mainly was from retrospective cohorts, which bears the risk of selection bias. Moreover, all development studies had a high RoB in the analysis domain. The nomograms might have been fitted to the characteristics of the development cohorts instead of a generalizable patient collective. In addition, no nomogram considered competing risks, which can cause an overestimation of the true event rate (57). Unfortunately, these limitations affect the reliability of the nomograms’ outcomes.

The meta-analyses identified several reliable nomogram predictors. Advanced pathological T-stage (≥pT3) predicted OS. Pathological T-stage (≥pT2), age, and LVI predicted CSS. LVI predicted RFS. These predictors were already known as individual risk factors (3, 58, 59). However, we elaborated that they retain their validity when combined, such as in a nomogram. Integrating novel biomarkers, reflecting the biological and clinical behavior of the tumor’s environment, could further improve the nomograms’ accuracy. So far, they have barely been considered.

The meta-analyses identified moderate discrimination accuracy for nomograms predicting OS, RFS, and IVR. Further, it identified good discrimination accuracy for nomograms predicting CSS. Because it was impossible to pool the c-Index of each nomogram separately, we could not identify the most accurate nomogram to be used in clinical practice. Instead, our analyses demonstrated the overall potential of postoperative prognostic UTUC nomograms, which justifies the effort for further research.

This systematic review highlights the critical absence of external validation studies, limiting nomograms’ applicability and uninformed use. Furthermore, it outlines that information on the clinical utility is scarce. Whether patients benefit from using nomograms remains unreported. However, improvements in postoperative risk stratification are urgently needed. Although the POUT trial and, most recently, Checkmate-274 demonstrated improved disease-free survival with adjuvant systemic therapy (platinum-based chemotherapy; nivolumab) in high-risk patients (10, 56), IMvigor010 failed to demonstrate any benefit (atezolizumab) (11). Further, the study raised concerns about postoperative TNM-based risk stratification (12). Therefore, future studies should focus on assessing the nomograms’ clinical utility and whether they can identify patients most suitable for adjuvant treatment.

This systematic review outlined similarities and differences between postoperative prognostic UTUC nomograms. Further, it provides physicians with a reference tool, enabling them to choose nomograms based on their individual needs and easily implement nomograms into clinical practice. For example, when searching for a nomogram predicting the five-year CSS following RNU, physicians can decide between the nomograms of Cha EK et al. (27) and Yates DR. et al. (43), as both have been externally validated. Further, they can decide whether to choose a nomogram taking the effects of adjuvant chemotherapy into account. As the last step, they can check whether the patient’s characteristics align with the patient cohort used for nomogram development or validation and whether the predictors are readily available. The reference tool will promote the widespread use of nomograms in postoperative UTUC patient counseling.

This study is the first systematic review summarizing postoperative prognostic UTUC nomograms. We set a standard for study quality, excluding all without internal validation data nor presenting discrimination and calibration accuracy. Although we used this most rigorous method, the approach could have missed potential nomograms. We estimated the nomograms’ and predictors’ overall predictive value by summarizing the c-Index and the coefficient Beta. Nevertheless, we could not estimate the overall nomogram calibration because the observed/expected rates were missing. However, calibration is essential to assess the benefit for clinical practice (60). We accounted for heterogeneity within the meta-analyses by stratifying nomograms into distinct groups. As a result, the number of studies included per analysis was low. Moreover, despite contacting all the corresponding authors in case of lacking results descriptions, our analyses were limited by missing values.

## 5 Conclusions

Despite a moderate to good discrimination accuracy, severe heterogeneity discourages the uninformed use of postoperative prognostic UTUC nomograms. For nomograms to become of value in a generalizable population, future research must invest in external validation and assessment of clinical utility. Meanwhile, this systematic review serves as a reference tool for physicians choosing nomograms based on individual needs.

## Data Availability Statement

The original contributions presented in the study are included in the article/**Supplementary Material**. Further inquiries can be directed to the corresponding author.

## Author Contributions

MP contributed to protocol/project development/management, data collection and management, data analysis and interpretation, and manuscript writing/editing. FK contributed to data collection and management, data interpretation, and manuscript writing/editing. AA, DD, EL, EX, FS, FQ, HM, LL, MRi, MRo, NS, PIK, PR, RSM, TK, TY, VM, and YL contributed to data interpretation and manuscript writing/editing. SFS contributed to protocol/project development/management, data interpretation, and manuscript writing/editing. BP contributed to protocol/project development/management, data collection and management, data interpretation, and manuscript writing/editing. All authors contributed to the article and approved the submitted version.

## funding

The author PR is supported by a EUSP Scholarship from the European Association of Urology.

## Conflict of Interest

The authors declare that the research was conducted in the absence of any commercial or financial relationships that could be construed as a potential conflict of interest.

The reviewer DE declared a shared affiliation with the author EL to the handling editor at the time of review.

## Publisher’s Note

All claims expressed in this article are solely those of the authors and do not necessarily represent those of their affiliated organizations, or those of the publisher, the editors and the reviewers. Any product that may be evaluated in this article, or claim that may be made by its manufacturer, is not guaranteed or endorsed by the publisher.

## Supplementary Material

Supplementary Table 1 Supplementary Table S1 lists the data extracted from the original publications.

Supplementary Table 2 Supplementary Table S2 lists the patient characteristics of nomogram development and validation cohorts in detail.

Supplementary Table 3 Supplementary Table S3 lists the predictors included within each nomogram in detail.

Supplementary Table 4 Supplementary Table S4 summarizes the predictors most frequently used with each nomogram group

Supplementary Table 5 Supplementary Table S5 gives a detailed overview of the nomograms’/studies’ RoB, giving RoB and applicability for each domain.

Supplementary Figure 1 Summary forest plot of nomogram group A predictor meta-analyses: The forest plot lists the results of individual meta-analyses. For each meta-analysis, the predictor’s name, the statistical approach, the number of values included (n), the prediction interval (lower limit – upper limit), and the c-Index summary estimate (estimate and 95% CIs) are given.

Supplementary Figure 2 Summary forest plot of nomogram group B1 predictor meta-analyses: The forest plot lists the results of individual meta-analyses. For each meta-analysis, the predictor’s name, the statistical approach, the number of values included (n), the prediction interval (lower limit – upper limit), and the c-Index summary estimate (estimate and 95% CIs) are given.

Supplementary Figure 3 Summary forest plot of nomogram group B2 predictor meta-analyses: The forest plot lists the results of individual meta-analyses. For each meta-analysis, the predictor’s name, the statistical approach, the number of values included (n), the prediction interval (lower limit – upper limit), and the c-Index summary estimate (estimate and 95% CIs) are given.

Supplementary Figure 4 Summary forest plot of nomogram group B3 predictor meta-analyses: The forest plot lists the results of individual meta-analyses. For each meta-analysis, the predictor’s name, the statistical approach, the number of values included (n), the prediction interval (lower limit – upper limit), and the c-Index summary estimate (estimate and 95% CIs) are given.

Supplementary Figure 5 Summary forest plot of nomogram group C predictor meta-analyses: The forest plot lists the results of individual meta-analyses. For each meta-analysis, the predictor’s name, the statistical approach, the number of values included (n), the prediction interval (lower limit – upper limit), and the c-Index summary estimate (estimate and 95% CIs) are given.

## Abbreviations

CI, Confidence Interval; CSS, Cancer-Specific Survival; HR, Hazard Ratio; IVR, Intravesical Recurrence; LVI, Lymphovascular Invasion; OS, Overall Survival; PI, Prediction Interval; PROBAST, Prediction model Risk Of Bias ASessment Tool; RFS, Recurrence-Free Survival; RoB, Risk of Bias; SE, Standard Error; UTUC, Upper Tract Urothelial Carcinoma.

## References

[1] Siegel RL Miller KD Fuchs HE Jemal A . Cancer Statistics, 2021. CA Cancer J Clin (2021) 71(1):7–33. doi: 10.3322/caac.21654 33433946

[2] Seisen T Peyronnet B Dominguez-Escrig JL Bruins HM Yuan CY Babjuk M . Oncologic Outcomes of Kidney-Sparing Surgery Versus Radical Nephroureterectomy for Upper Tract Urothelial Carcinoma: A Systematic Review by the EAU Non-Muscle Invasive Bladder Cancer Guidelines Panel. Eur Urol (2016) 70(6):1052–68. doi: 10.1016/j.eururo.2016.07.014 27477528

[3] Margulis V Shariat SF Matin SF Kamat AM Zigeuner R Kikuchi E . Outcomes of Radical Nephroureterectomy: A Series From the Upper Tract Urothelial Carcinoma Collaboration. Cancer (2009) 115(6):1224–33. doi: 10.1002/cncr.24135 19156917

[4] Favaretto RL Shariat SF Savage C Godoy G Chade DC Kaag M . Combining Imaging and Ureteroscopy Variables in a Preoperative Multivariable Model for Prediction of Muscle-Invasive and non-Organ Confined Disease in Patients With Upper Tract Urothelial Carcinoma. BJU Int (2012) 109(1):77–82. doi: 10.1111/j.1464-410X.2011.10288.x 21631698 PMC4319659

[5] Chromecki TF Cha EK Fajkovic H Margulis V Novara G Scherr DS . The Impact of Tumor Multifocality on Outcomes in Patients Treated With Radical Nephroureterectomy. Eur Urol (2012) 61(2):245–53. doi: 10.1016/j.eururo.2011.09.017 21975249

[6] Favaretto RL Shariat SF Chade DC Godoy G Adamy A Kaag M . The Effect of Tumor Location on Prognosis in Patients Treated With Radical Nephroureterectomy at Memorial Sloan-Kettering Cancer Center. Eur Urol (2010) 58(4):574–80. doi: 10.1016/j.eururo.2010.07.003 PMC4174409 20637540

[7] Rouprêt M Babjuk M Burger M Capoun O Cohen D Compérat EM . European Association of Urology Guidelines on Upper Urinary Tract Urothelial Carcinoma: 2020 Update. Eur Urol (2021) 79(1):62–79. doi: 10.1016/j.eururo.2020.05.042 32593530

[8] Birtle A Johnson M Chester J Jones R Dolling D Bryan RT . Adjuvant Chemotherapy in Upper Tract Urothelial Carcinoma (the POUT Trial): A Phase 3, Open-Label, Randomised Controlled Trial. Lancet (2020) 395(10232):1268–77. doi: 10.1016/S0140-6736(20)30415-3 PMC7181180 32145825

[9] Birtle AJ Chester JD Jones RJ Jenkins B Johnson M Catto JW . Updated Outcomes of POUT: A Phase III Randomized Trial of Peri-Operative Chemotherapy Versus Surveillance in Upper Tract Urothelial Cancer (UTUC). J Clin Oncol (2021) 39(6_suppl):455–5. doi: 10.1200/JCO.2021.39.6_suppl.455

[10] Bajorin DF Witjes JA Gschwend JE Schenker M Valderrama BP Tomita Y . Adjuvant Nivolumab Versus Placebo in Muscle-Invasive Urothelial Carcinoma. N Engl J Med (2021) 384(22):2102–14. doi: 10.1056/NEJMoa2034442 PMC8215888 34077643

[11] Bellmunt J Hussain M Gschwend JE Albers P Oudard S Castellano D . Adjuvant Atezolizumab Versus Observation in Muscle-Invasive Urothelial Carcinoma (IMvigor010): A Multicentre, Open-Label, Randomised, Phase 3 Trial. Lancet Oncol (2021) 22(4):525–37. doi: 10.1016/S1470-2045(21)00004-8 PMC8495594 33721560

[12] Powles T Assaf ZJ Davarpanah N Banchereau R Szabados BE Yuen KC . ctDNA Guiding Adjuvant Immunotherapy in Urothelial Carcinoma. Nature (2021) 595(7867):432–7. doi: 10.1038/s41586-021-03642-9 34135506

[13] Iasonos A Schrag D Raj GV Panageas KS . How to Build and Interpret a Nomogram for Cancer Prognosis. J Clin Oncol (2008) 26(8):1364–70. doi: 10.1200/JCO.2007.12.9791 18323559

[14] Lughezzani G Briganti A Karakiewicz PI Kattan MW Montorsi F Shariat SF . Predictive and Prognostic Models in Radical Prostatectomy Candidates: A Critical Analysis of the Literature. Eur Urol (2010) 58(5):687–700. doi: 10.1016/j.eururo.2010.07.034 20727668 PMC4119802

[15] Shariat SF Kattan MW Vickers AJ Karakiewicz PI Scardino PT . Critical Review of Prostate Cancer Predictive Tools. Future Oncol (2009) 5(10):1555–84. doi: 10.2217/fon.09.121 PMC2933457 20001796

[16] Debray TP Damen JA Snell KI Ensor J Hooft L Reitsma JB . A Guide to Systematic Review and Meta-Analysis of Prediction Model Performance. BMJ (2017) 356:i6460. doi: 10.1136/bmj.i6460 28057641

[17] Moons KG de Groot JA Bouwmeester W Vergouwe Y Mallett S Altman DG . Critical Appraisal and Data Extraction for Systematic Reviews of Prediction Modelling Studies: The CHARMS Checklist. PloS Med (2014) 11(10):e1001744. doi: 10.1371/journal.pmed.1001744 25314315 PMC4196729

[18] Page MJ McKenzie JE Bossuyt PM Boutron I Hoffmann TC Mulrow CD . The PRISMA 2020 Statement: An Updated Guideline for Reporting Systematic Reviews. BMJ (2021) 372:n71. doi: 10.1136/bmj.n71 33782057 PMC8005924

[19] Altman DG Bland JM . How to Obtain the Confidence Interval From a P Value. BMJ (2011) 343:d2090. doi: 10.1136/bmj.d2090 21824904

[20] Debray TP Damen JA Riley RD Snell K Reitsma JB Hooft L . A Framework for Meta-Analysis of Prediction Model Studies With Binary and Time-To-Event Outcomes. Stat Methods Med Res (2019) 28(9):2768–86. doi: 10.1177/0962280218785504 PMC6728752 30032705

[21] Moons KGM Wolff RF Riley RD Whiting PF Westwood M Collins GS . PROBAST: A Tool to Assess Risk of Bias and Applicability of Prediction Model Studies: Explanation and Elaboration. Ann Intern Med (2019) 170(1):W1–W33. doi: 10.7326/M18-1377 30596876

[22] Harrell FE Califf RM Pryor DB Lee KL Rosati RA . Evaluating the Yield of Medical Tests. JAMA (1982) 247(18):2543–6. doi: 10.1001/jama.247.18.2543 7069920

[23] George B Seals S Aban I . Survival Analysis and Regression Models. J Nucl Cardiol (2014) 21(4):686–94. doi: 10.1007/s12350-014-9908-2 PMC4111957 24810431

[24] Balduzzi S Rücker G Schwarzer G . How to Perform a Meta-Analysis With R: A Practical Tutorial. Evidence-Based Ment Health (2019) 22:153–60. doi: 10.1136/ebmental-2019-300117 PMC10231495 31563865

[25] Viechtbauer W . Conducting Meta-Analyses in R With the Metafor Package. J Stat Software (2010) 36(3):1–48. doi: 10.18637/jss.v036.i03

[26] Abdul-Muhsin H De Lucia N Singh V Faraj K Rose K Cha S . Outcome Prediction Following Radical Nephroureterectomy for Upper Tract Urothelial Carcinoma. Urol Oncol (2021) 39(2):133.e9–133.e16. doi: 10.1016/j.urolonc.2020.08.021 33069555

[27] Cha EK Shariat SF Kormaksson M Novara G Chromecki TF Scherr DS . Predicting Clinical Outcomes After Radical Nephroureterectomy for Upper Tract Urothelial Carcinoma. Eur Urol (2012) 61(4):818–25. doi: 10.1016/j.eururo.2012.01.021 22284969

[28] Chen J Zhong W Yang M Hou W Wang X Xia K . Development and Validation of a PD-L1/PD-1/CD8 Axis-Based Classifier to Predict Cancer Survival of Upper Tract Urothelial Carcinoma After Radical Nephroureterectomy. Cancer Immunol Immunother (2021) 70(9):2657–68. doi: 10.1007/s00262-020-02827-x PMC10992229 33606065

[29] Chen X Ji H Wang J Zhao G Zheng B Niu Z . Prognostic Value of the Preoperative Plasma D-Dimer Levels in Patients With Upper Tract Urothelial Carcinoma in a Retrospective Cohort Study. Onco Targets Ther (2020) 13:5047–55. doi: 10.2147/OTT.S254514 PMC7292253 32606727

[30] Fang D Singla N Bao Z Jafri SM Su X Cao Z . The Significance of Preoperative Serum Sodium and Hemoglobin in Outcomes of Upper Tract Urothelial Carcinoma: Multi-Center Analysis Between China and the United States. Cancer Manag Res (2020) 12:9825–36. doi: 10.2147/CMAR.S267969 PMC7549885 33116841

[31] Hou G Zheng Y Zhang L Lai D Wang F Li X . Development and Validation of a Prognostic Nomogram for Patients With Intravesical Recurrence After Radical Nephroureterectomy for Non-Metastatic Upper Tract Urothelial Carcinoma. World J Urol (2020) 38(8):1969–75. doi: 10.1007/s00345-019-02985-3 31654221

[32] Jeldres C Sun M Lughezzani G Isbarn H Shariat SF Widmer H . Highly Predictive Survival Nomogram After Upper Urinary Tract Urothelial Carcinoma. Cancer (2010) 116(16):3774–84. doi: 10.1002/cncr.25122 20564085

[33] Kim M Moon KC Choi WS Jeong CW Kwak C Kim HH . Prognostic Value of Systemic Inflammatory Responses in Patients With Upper Urinary Tract Urothelial Carcinoma. World J Urol (2015) 33(10):1439–57. doi: 10.1007/s00345-015-1484-9 25600022

[34] Kim SH Song MK Hong B Kang SH Jeong BC Ku JH . Developing a Prediction Model for Disease-Free Survival From Upper Urinary Tract Urothelial Carcinoma in the Korean Population. Cancer Med (2019) 8(11):4967–75. doi: 10.1002/cam4.2382 PMC6718545 31283107

[35] Krabbe LM Eminaga O Shariat SF Hutchinson RC Lotan Y Sagalowsky AI . Postoperative Nomogram for Relapse-Free Survival in Patients With High Grade Upper Tract Urothelial Carcinoma. J Urol (2017) 197(3 Pt 1):580–9. doi: 10.1016/j.juro.2016.09.078 27670916

[36] Ku JH Moon KC Jung JH Jeong SH Kwak C Kim HH . External Validation of an Online Nomogram in Patients Undergoing Radical Nephroureterectomy for Upper Urinary Tract Urothelial Carcinoma. Br J Cancer (2013) 109(5):1130–6. doi: 10.1038/bjc.2013.462 PMC3778306 23949152

[37] Lai S Long X Wu P Liu J Seery S Hou H . Developing a Nomogram for Predicting Intravesical Recurrence After Radical Nephroureterectomy: A Retrospective Cohort Study of Mainland Chinese Patients. Jpn J Clin Oncol (2021) 51(7):1132–41. doi: 10.1093/jjco/hyab017 33634310

[38] Lai S Wu P Diao T Seery S Liu J Hou H . Does Xylinas' Nomogram Accurately Predict Intravesical Recurrence Risk After Radical Nephroureterectomy for Primary Upper Urinary Tract Urothelial Carcinoma When Applied to Asian Populations? Jpn J Clin Oncol (2021) 51(3):469–77. doi: 10.1093/jjco/hyaa138 32734304

[39] Rouprêt M Hupertan V Seisen T Colin P Xylinas E Yates DR . Prediction of Cancer Specific Survival After Radical Nephroureterectomy for Upper Tract Urothelial Carcinoma: Development of an Optimized Postoperative Nomogram Using Decision Curve Analysis. J Urol (2013) 189(5):1662–9. doi: 10.1016/j.juro.2012.10.057 23103802

[40] Seisen T Colin P Hupertan V Yates DR Xylinas E Nison L . Postoperative Nomogram to Predict Cancer-Specific Survival After Radical Nephroureterectomy in Patients With Localised and/or Locally Advanced Upper Tract Urothelial Carcinoma Without Metastasis. BJU Int (2014) 114(5):733–40. doi: 10.1111/bju.12631 24447471

[41] Wang M Ren X Wang G Sun X Tang S Zhang B . Construction of a Survival Prediction Model for High-And Low -Grade UTUC After Tumor Resection Based on "SEER Database": A Multicenter Study. BMC Cancer (2021) 21(1):999. doi: 10.1186/s12885-021-08742-3 34493229 PMC8424798

[42] Xylinas E Kluth L Passoni N Trinh QD Rieken M Lee RK . Prediction of Intravesical Recurrence After Radical Nephroureterectomy: Development of a Clinical Decision-Making Tool. Eur Urol (2014) 65(3):650–8. doi: 10.1016/j.eururo.2013.09.003 24070577

[43] Yates DR Hupertan V Colin P Ouzzane A Descazeaud A Long JA . Cancer-Specific Survival After Radical Nephroureterectomy for Upper Urinary Tract Urothelial Carcinoma: Proposal and Multi-Institutional Validation of a Post-Operative Nomogram. Br J Cancer (2012) 106(6):1083–8. doi: 10.1038/bjc.2012.64 PMC3304431 22374463

[44] Zeng S Dai L Yang J Gao X Yu X Ren Q . Development and External Validation of a Nomogram Predicting Prognosis of Upper Tract Urothelial Carcinoma After Radical Nephroureterectomy. Urol Oncol (2019) 37(4):290.e17–290.e24. doi: 10.1016/j.urolonc.2018.12.027 30630733

[45] Zhang GL Zhou W . A Model for the Prediction of Survival in Patients With Upper Tract Urothelial Carcinoma After Surgery. Dose Response (2019) 17(4):1559325819882872. doi: 10.1177/1559325819882872 31662711 PMC6794662

[46] Zhang X Bu R Liu Z Wu B Bai S . Development and Validation of a Model for Predicting Intravesical Recurrence in Organ-Confined Upper Urinary Tract Urothelial Carcinoma Patients After Radical Nephroureterectomy: A Retrospective Study in One Center With Long-Term Follow-Up. Pathol Oncol Res (2020) 26(3):1741–8. doi: 10.1007/s12253-019-00748-4 31643022

[47] Li C Yang J Xu F Han D Zheng S Kaaya RE . A Prognostic Nomogram for the Cancer-Specific Survival of Patients With Upper-Tract Urothelial Carcinoma Based on the Surveillance, Epidemiology, and End Results Database. BMC Cancer (2020) 20(1):534. doi: 10.1186/s12885-020-07019-5 32513124 PMC7282122

[48] Li C Han D Huang Q Xu F Zheng S Li X . Competing-Risks Nomogram for Predicting Cancer-Specific Death in Upper Tract Urothelial Carcinoma: A Population-Based Analysis. BMJ Open (2021) 11(7):e048243. doi: 10.1136/bmjopen-2020-048243 PMC8291317 34281927

[49] Qi F Wei X Zheng Y Sha Y Lu Y Li X . Nomograms to Predict Overall and Cancer-Specific Survival in Patients With Upper Tract Urothelial Carcinoma: A Large Population-Based Study. Transl Androl Urol (2020) 9(3):1177–91. doi: 10.21037/tau.2020.03.28 PMC7354328 32676401

[50] Wu J Chen S Wu X Mao W Wang Y Xu B . Trends of Incidence and Prognosis of Upper Tract Urothelial Carcinoma. Bosn J Basic Med Sci (2021) 21(5):607–19. doi: 10.17305/bjbms.2020.5345 PMC8381214 33357210

[51] Siontis GC Tzoulaki I Castaldi PJ Ioannidis JP . External Validation of New Risk Prediction Models Is Infrequent and Reveals Worse Prognostic Discrimination. J Clin Epidemiol (2015) 68(1):25–34. doi: 10.1016/j.jclinepi.2014.09.007 25441703

[52] van Doeveren T van der Mark M van Leeuwen PJ Boormans JL Aben KKH . Rising Incidence Rates and Unaltered Survival Rates for Primary Upper Urinary Tract Urothelial Carcinoma: A Dutch Population-Based Study From 1993 to 2017. BJU Int (2021) 128(3):343–51. doi: 10.1111/bju.15389 PMC8453942 33690922

[53] Collà Ruvolo C Nocera L Stolzenbach LF Wenzel M Cucchiara V Tian Z . Incidence and Survival Rates of Contemporary Patients With Invasive Upper Tract Urothelial Carcinoma. Eur Urol Oncol (2021) 4(5):792–801. doi: 10.1016/j.euo.2020.11.005 33293235

[54] Shiga M Nagumo Y Chihara I Nitta S Kojo K Kimura T . Discrepancy Between Clinical and Pathological T Stages in Upper Urinary Tract Urothelial Carcinoma: Analysis of the Hospital-Based Cancer Registry Data in Japan. Int J Urol (2021) 28(8):814–9. doi: 10.1111/iju.14583 34013614

[55] Collà Ruvolo C Nocera L Stolzenbach LF Wenzel M Califano G Tian Z . Tumor Size Predicts Muscle-Invasive and Non-Organ-Confined Disease in Upper Tract Urothelial Carcinoma at Radical Nephroureterectomy. Eur Urol Focus (2021) S2405–4569. doi: 10.1016/j.euf.2021.03.003 33737024

[56] Leow JJ Chong YL Chang SL Valderrama BP Powles T Bellmunt J . Neoadjuvant and Adjuvant Chemotherapy for Upper Tract Urothelial Carcinoma: A 2020 Systematic Review and Meta-Analysis, and Future Perspectives on Systemic Therapy. Eur Urol (2021) 79(5):635–54. doi: 10.1016/j.eururo.2020.07.003 32798146

[57] Kim HT . Cumulative Incidence in Competing Risks Data and Competing Risks Regression Analysis. Clin Cancer Res (2007) 13(2 Pt 1):559–65. doi: 10.1158/1078-0432.CCR-06-1210 17255278

[58] Petrelli F Yasser Hussein MI Vavassori I Barni S . Prognostic Factors of Overall Survival in Upper Urinary Tract Carcinoma: A Systematic Review and Meta-Analysis. Urology (2017) 100:9–15. doi: 10.1016/j.urology.2016.07.036 27516121

[59] Liu W Sun L Guan F Wang F Zhang G . Prognostic Value of Lymphovascular Invasion in Upper Urinary Tract Urothelial Carcinoma After Radical Nephroureterectomy: A Systematic Review and Meta-Analysis. Dis Markers (2019) 2019:7386140. doi: 10.1155/2019/7386140 31565103 PMC6745116

[60] Moons KG Altman DG Reitsma JB Ioannidis JP Macaskill P Steyerberg EW . Transparent Reporting of a Multivariable Prediction Model for Individual Prognosis or Diagnosis (TRIPOD): Explanation and Elaboration. Ann Intern Med (2015) 162(1):W1–73. doi: 10.7326/M14-0698 25560730

