# Supplementary figures and images for: A Systematic Review and Meta-Analysis of Prognostic Nomograms After UTUC Surgery
Source: Front Oncol. 2022 Jul 1;12:907975. doi: 10.3389/fonc.2022.907975 (PMC9283688; doi:10.3389/fonc.2022.907975)

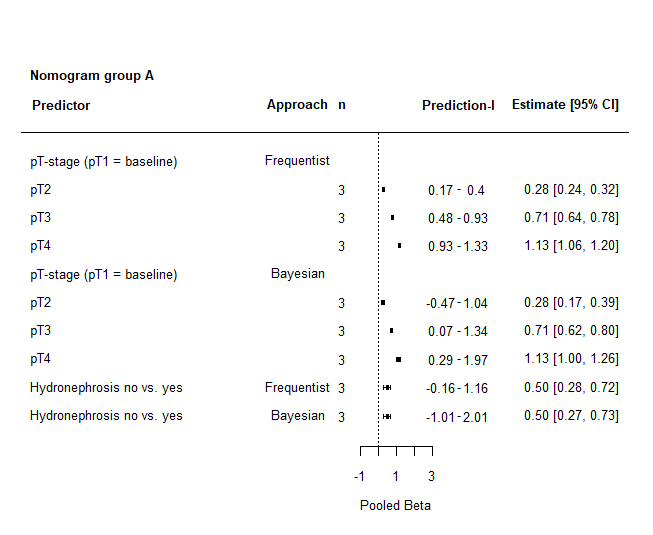

Supplement: Supplementary Figure 1 — Summary forest plot of nomogram group A predictor meta-analyses: The forest plot lists the results of individual meta-analyses. For each meta-analysis, the predictor’s name, the statistical approach, the number of values included (n), the prediction interval (lower limit – upper limit), and the c-Index summary estimate (estimate and 95% CIs) are given. [file Image_1.tif]

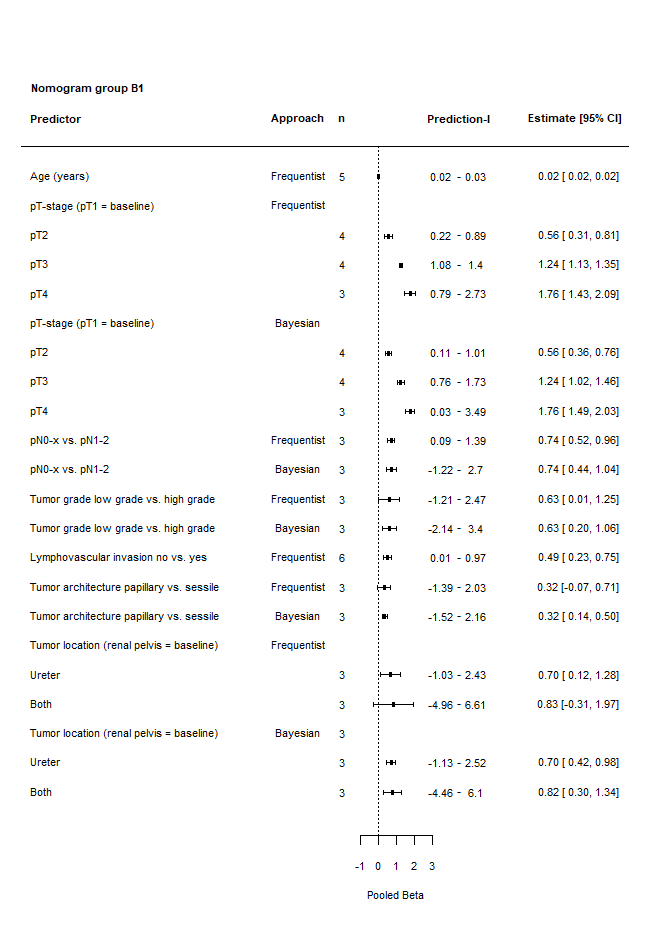

Supplement: Supplementary Figure 2 — Summary forest plot of nomogram group B1 predictor meta-analyses: The forest plot lists the results of individual meta-analyses. For each meta-analysis, the predictor’s name, the statistical approach, the number of values included (n), the prediction interval (lower limit – upper limit), and the c-Index summary estimate (estimate and 95% CIs) are given. [file Image_2.tif]

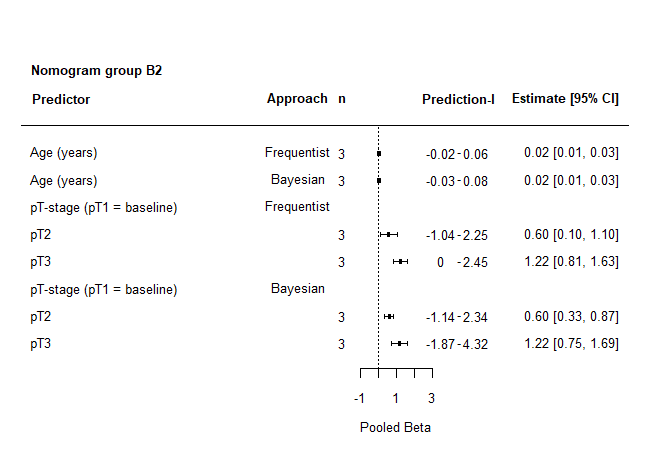

Supplement: Supplementary Figure 3 — Summary forest plot of nomogram group B2 predictor meta-analyses: The forest plot lists the results of individual meta-analyses. For each meta-analysis, the predictor’s name, the statistical approach, the number of values included (n), the prediction interval (lower limit – upper limit), and the c-Index summary estimate (estimate and 95% CIs) are given. [file Image_3.tif]

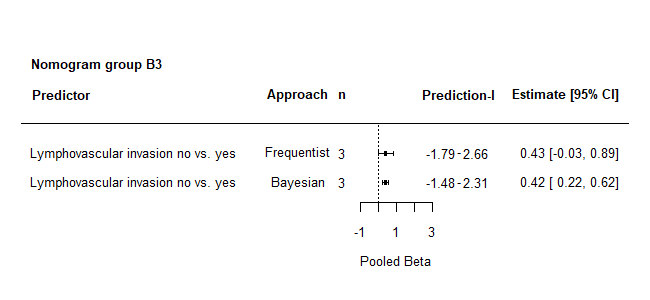

Supplement: Supplementary Figure 4 — Summary forest plot of nomogram group B3 predictor meta-analyses: The forest plot lists the results of individual meta-analyses. For each meta-analysis, the predictor’s name, the statistical approach, the number of values included (n), the prediction interval (lower limit – upper limit), and the c-Index summary estimate (estimate and 95% CIs) are given. [file Image_4.tif]

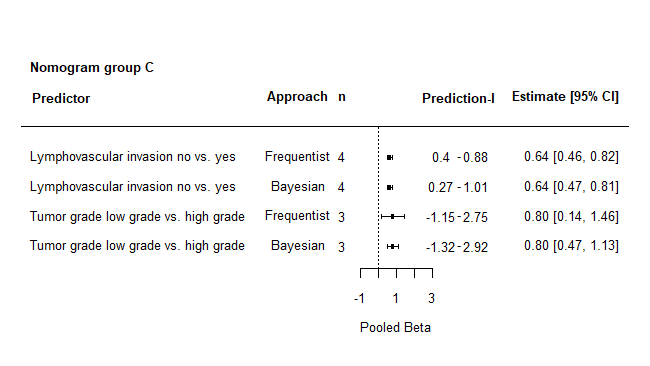

Supplement: Supplementary Figure 5 — Summary forest plot of nomogram group C predictor meta-analyses: The forest plot lists the results of individual meta-analyses. For each meta-analysis, the predictor’s name, the statistical approach, the number of values included (n), the prediction interval (lower limit – upper limit), and the c-Index summary estimate (estimate and 95% CIs) are given. [file Image_5.tif]
